# Cardiomyocyte Regeneration in Human Myocarditis

**DOI:** 10.3390/biomedicines12081814

**Published:** 2024-08-09

**Authors:** Andrea Frustaci, Eleonora Foglio, Federica Limana, Michele Magnocavallo, Emanuela Frustaci, Leonardo Lupacchini, Romina Verardo

**Affiliations:** 1 Cellular and Molecular Cardiology Lab, IRCCS L. Spallanzani, Via Portuense, 292, 00149 Rome, Italy; romina.verardo@inmi.it; 2 Technoscience, Parco Scientifico e Tecnologico Pontino, 04100 Latina, Italy; eleonora.foglio83@gmail.com (E.F.); emanuela.frustaci@outlook.it (E.F.); 3 Department of Human Sciences and Promotion of Quality of Life, San Raffaele University of Rome, IRCCS San Raffaele, 00163 Rome, Italy; federicalimana@gmail.com; 4 Arrhythmology Unit, Ospedale Isola Tiberina—Gemelli Isola, 00186 Rome, Italy; michelefg91@gmail.com; 5 Department of Molecular Medicine, Sapienza University of Rome, 00185 Rome, Italy; 6 MEBIC Consortium, IRCCS San Raffaele Roma, 00163 Rome, Italy; 7 Laboratory of Molecular and Cellular Neuroscience, IRCCS San Raffaele Roma, 00163 Rome, Italy; leonardo.lupacchini@sanraffaele.it

**Keywords:** myocarditis, newly generated cardiomyocytes, STAT3

## Abstract

Background: Newly generated cardiomyocytes (NGCs) concur with the recovery of human myocarditis occurring spontaneously in around 50% of cases. However, NGCs decline with age, and their modality of myocardial homing and integration are still unclear. Methods: We retrospectively assessed NGCs in 213 consecutive patients with endomyocardial biopsy denoting acute myocarditis, with normal coronaries and valves. Tissue samples were processed for histology (H&E), immunohistochemistry for the evaluation of inflammatory infiltrates, immunostaining for alpha-sarcomeric-actin, junctional connexin-43, Ki-67, and phosphorylated STAT3 (p-STAT3), and Western blot (WB) for HMGB1. Frozen samples were analyzed using polymerase chain reaction (PCR) for cardiotropic viruses. Controls included 20 normal surgical biopsies. Results: NGCs were defined as small myocytes (diameter < 10 µm) with nuclear positivity to Ki-67 and p-STAT3 and positive immunostaining for cytoplasmic α-sarcomeric actin and connexin-43. Their number/mm^2^ in relation to age and pathway of integration was evaluated. NGCs crossed the membrane and grew integrated within the empty necrotic myocytes. NGC mean diameter was 6.6 ± 3.34 vs. 22.5 ± 3.11 µm adult cells; their number, in comparison to LVEF, was 86.3 ± 10.3/mm^2^ in patients between 18 and 40 years, 50.4 ± 13.8/mm^2^ in those between 41 and 60, and 15.1 ± 5.7/mm^2^ in those between 61 and 80. Control NGCs’ mean diameter was 0.2 ± 0.2 mm^2^. PCR was positive for viral genomes in 16% of cases; NGCs were not statistically different in viral and non-viral myocarditis. WB analysis revealed a higher expression of HMGB1 in myocarditis compared to myocardial controls. Conclusions: NGCs are constantly recognizable in acute human myocarditis. Their number declines with age. Their integration within necrotic myocytes allows for the preservation of the cardiac structure and function.

## 1. Introduction

Recovery of cardiac function in human myocarditis is a common event and may spontaneously occur in as many as 50% of patients [1]. Various mechanisms contribute, including the decline of cell death and the resynthesis of contractile proteins and then cell proliferation [2]. Specifically, apoptosis and necrosis of cardiomyocytes have been found to decrease by 85% and 62%, respectively, in patients with virus-negative myocarditis responding to immunosuppressive therapy. The myofibrillar content of myocardiocytes evaluated through ultrastructural morphometry was shown to increase by 33% in myocarditis subjects recovering and decline in those with further functional impairment. Finally, the level of myocyte replication has been reported to increase by 43% for Ki-67 and by 38% for mini-chromosome maintenance 5 (MCM5)-positive cells after 6 months of successful immunosuppression [2]. The mechanisms through which newly generated cardiomyocytes (NGCs) are recruited and properly integrated into the myocardium are still unknown.

Miyawaki et al. showed that the signal transducer and activator of transcription 3 (STAT3) is involved in cell cycle re-entry of adult cardiomyocytes in an experimental autoimmune myocarditis (EAM) model [3]. In their study, 3 weeks after EAM induction through immunization with peptides derived from mouse α-myosin heavy chain (α-MHC), cardiac tissue was severely injured by inflammatory cell infiltration. Cardiac tissue restoration has been attributed to cell cycle re-entry of pre-existing mononucleated cardiomyocytes through the activation of STAT3. This finding was further supported by the enhancement in fibrosis detected in STAT3 cardiomyocyte-specific KO mice following 5 weeks of immunization. Importantly, a critical role of STAT3 has also been demonstrated in zebrafish and neonatal mouse cardiomyocyte proliferation [4,5]. Specifically, using a zebrafish model of ventricular ablation, early myocardial activation of the Janus kinase signal transducer 1 and STAT3 (Jak1/STAT3) downstream mediators by tissue damage stimulated the production of the protein Relaxin 3A (Rln3a), necessary for cardiomyocyte proliferation and heart regeneration [5]. It is also interesting to note that different studies have indicated pro-regenerative roles of inflammatory cytokines in other terminally differentiated tissues, like the zebrafish brain [6]. Accordingly, the study by Han et al. showed that IL-6 was essential for heart regeneration in neonatal mice and, consistently, cardiomyocyte-specific ablation of STAT3, the major downstream effector of IL-6 signaling, impaired cardiomyocyte proliferative response after apical resection [4].

The IL-6/STAT3 signaling pathway is also essential for stem/progenitor cell proliferation [6,7], and high mobility group box 1 (HMGB1) is able to trigger the activation of this pathway [8,9]. In particular, HMGB1, produced by stressed cardiomyocytes, induces the secretion of IL6, which activates the phosphorylation of STAT3. Furthermore, the influence of aging on this process is still unclear.

We report the assessment of NGCs in a large population of patients with an age range between 18 and 80 years and a biopsy-proven myocarditis of recent (≤4 weeks) onset. NGCs are identified through immunostaining positivity to nuclear markers of cardiomyocyte replication (Ki67 and p-STAT3) and based on evidence of intracytoplasmic synthesis of contractile material (alpha-sarcomeric-actin and connexin-43). Their number of 2 mm is correlated with patients’ age. Finally, the mechanism of integration of Ngcs into necrotic myocardiocytes is analyzed.

## 2. Methods

We retrospectively enrolled consecutive patients (age range 18–80 years) from January 2010 to June 2021 with a left ventricular endomyocardial biopsy diagnostic for active myocarditis of recent (≤1 month) onset of symptoms, including fever, chest pain, and increased troponin I. The 213 enrolled patients with acute myocarditis were divided into 3 groups depending on their age. Group 1 (G1) included patients aged 18–40 years, Group 2 (G2) included patients aged 41–60 years, and Group 3 (G3) included patients aged 61–80 years in order to investigate the proliferative reserve of cardiomyocytes in relation to age.

### 2.1. Cardiac Studies

All subjects before cardiac biopsy underwent an extensive preliminary study, including serum troponin I, 2D echo showing normal valves, and cardiac magnetic resonance (CMR) suggesting myocardial edema and/or fibrosis. Echocardiographic parameters were determined according to established criteria [10].

Specifically, the left ventricular ejection fraction (LVEF) was determined using the apical 4- and 2-chamber views from three distinct cardiac cycles through the modified Simpson’s method.

CMR imaging was performed using a 1.5 Tesla scanner (Avanto Siemens, Erlangen, Germany).

The standard protocol for cardiac magnetic resonance included (i) cine magnetic resonance images captured in the short-axis, 2-chamber, and 4-chamber views during breath-holds; (ii) black blood T2-weighted short-tau inversion recovery images obtained on short-axis planes spanning the entire left ventricle with 6 to 8 consecutive breath-holds for the detection of myocardial edema; and (iii) late gadolinium-enhanced imaging conducted 15 min post-injection of 0.2 nmol/kg of gadoterate meglumine, where a signal intensity value 2 standard deviations above the mean signal intensity of the normal myocardium was indicative of myocardial fibrosis. Analysis of functional and morphological data was carried out in accordance with the Lake Louise criteria [11], where a CMR diagnosis of myocarditis was suggested by the presence of at least two of the following criteria: early enhancement (hyperemia as an equivalent of vasodilation), edema on T2-weighted/STIR sequences, and late gadolinium enhancement in a non-ischemic pattern.

### 2.2. Endomyocardial Biopsy Studies

Cardiac catheterization with left ventricular and coronary angiography was performed in all patients. A biopsy of the endomyocardium, consisting of 4 to 8 samples per patient, was carried out in the septal-apical area of the left ventricle [12].

### 2.3. Histology, Immunohistochemistry, and Assessment of NGCs

For histological analysis, the endomyocardial samples were fixed in 10% buffered formalin and paraffin-embedded. Subsequently, five-micron-thick sections were stained using hematoxylin and eosin (H&E).

The diagnosis of myocarditis histologically included the presence of leukocyte infiltrates alongside damage to neighboring myocytes, following the Dallas criteria and confirmed via immunohistochemistry [13]. Immunohistochemistry for CD3, CD20, CD43, CD45RO, and CD68 (all from Dako, Carpinteria, CA, USA) was carried out to characterize the inflammatory infiltrates. A diagnosis of myocarditis was established if an inflammatory infiltrate of ≥14 leucocytes/mm^2^, including up to 4 monocytes/mm^2^, with CD3-positive T-lymphocytes of ≥7 cells/mm^2^, was present, in association with signs of degeneration or necrosis in the nearby cardiomyocytes.

NGCs were defined as small myocytes (diameter < 10 µm) with positive cytoplasm immunostaining for α-sarcomeric actin, cell membrane expressing connexin-43 and nuclear positivity to Ki-67 and p-STAT3. To analyze the possible influence of aging on NGC production, the number of NGCs was compared in groups of 20 patients of advancing age. In particular, the number of NGCs was determined in patients between 10 and 40 years (Group 1—G1), in those between 41 and 60 years (Group 2—G2), and in those between 61 and 80 years (Group 3—G3). Control NGCs were obtained from 20 normal surgical biopsies.

### 2.4. Real-Time PCR

At baseline, real-time PCR [14] was conducted in all patients to detect the presence of common cardiotropic viruses, such as adenovirus, enterovirus, influenza A and B viruses, Epstein–Barr virus, parvovirus B19, hepatitis C virus, cytomegalovirus, human herpesvirus 6, and herpes simplex virus type 1 and 2, aiming to identify the etiology of myocarditis.

### 2.5. Western Blot Assay

Proteins extracted from endomyocardial biopsies were subjected to homogenization and extraction utilizing Lysis buffer, which consists of 50 mM Tris-HCl pH 7.4, 5 mM EDTA, 250 mM sodium chloride (NaCl), and 0.1% Triton^®^ X-100. This buffer was freshly supplemented with protease and phosphatase inhibitors, including 0.1 mMol/L Dithiothreitol (DTT), 50 mM sodium fluoride (NaF), 0.1 mM sodium ortho-vanadate (Na_3_VO_4_), 1 mM phenylmethyl–sulfonyl fluoride (PMSF), and 1× protease inhibitor cocktail, all of which were purchased from Sigma-Aldrich_MERCK, St. Louis, MO, USA. The concentration of proteins was quantified through a Bradford assay (Bio-Rad, Hercules, CA, USA) using the 96-well plater reader Glo-Max^®^-Multi Detection System (Promega Corporation, Madison, WI, USA).

Subsequently, equal amounts of total cellular proteins (30 µg/lane) were boiled for 5 min at 95 °C, followed by resolution using denaturizing SDS-polyacrylamide gel electrophoresis, and transferred onto a 0.45 µm nitrocellulose membrane (Bio-Rad, Hercules, CA, USA).

After blocking with 5% non-fat dry milk (Bio-Rad, Hercules, CA, USA), membranes were incubated with a mouse monoclonal anti-HMGB1 primary antibody (1:500; MA5-17278, Invitrogen, Waltham, MA, USA) and a mouse monoclonal anti-GAPDH primary antibody (1:750; sc-137179, Santa Cruz Biotechnology Inc., Dallas, TX, USA) overnight at 4 °C, followed by appropriated horseradish peroxidase-coupled secondary antibodies and developed using a chemiluminescence-based detection system (Lite Ablot^®^ TURBO; EuroClone, Milan, Italy). Densitometric analysis of the bands, relative to GAPDH, was determined using ImageJ Software v1.51 (National Institutes of Health, Bethesda, MD, USA).

### 2.6. Immunofluorescence

For immunofluorescence staining, human endomyocardial paraffin-embedded sections (5 μm thickness) were dewaxed in xylene, hydrated, and rinsed in phosphate-buffered saline (PBS). Antigen retrieval was performed using, alternatively, 1 mM ethylenediaminetetraacetic acid (EDTA) buffer (pH 8) or TRIS EDTA-citrate buffer (pH 7.8) at 98 °C for 20 min, depending on the antigen localization (nuclear or cytoplasmatic, respectively). After cooling, sections were permeabilized for 10 min with 0.1% Triton X-100 in PBS. After a 1 h block with antibody diluent with background-reducing components (Dako, Agilent, Santa Clara, CA, USA), sections were incubated in a humidified chamber at 4 °C overnight with the following primary antibodies: mouse monoclonal anti-ki67 (ready to use, Leica Biosystems, Milan, Italy), rabbit polyclonal anti-connexin-43 (1:750, Sigma-Aldrich, MERCK, St. Louis, MO, USA), or rabbit monoclonal anti-phospho STAT3 (1:200, Cell Signaling Technology, Danvers, MA, USA). Afterwards, slides were washed with PBS 3 times (5 min/wash) and incubated for 1 h with goat anti-mouse IgG Alexa Fluor 488 or goat anti-rabbit IgG Alexa Fluor 555 fluorescent secondary antibodies (1:200, Invitrogen, Thermo Fisher Scientific, Carlsbad, CA, USA). After that, to perform double staining, biopsies were further incubated with an anti α-sarcomeric actin primary antibody (rabbit or mouse monoclonal, respectively, for double staining with Ki-67 or connexin-43) (1:100 or 1:50, Sigma-Aldrich, MERCK, St. Louis, MO, USA) for 1 h at 37 °C, followed by a properly combined fluorescent secondary antibody incubation.

Finally, sections were washed with PBS 3 times (5 min/wash), and nuclei were stained using DAPI (1:10,000, 5 min; Molecular Probes, Eugene, OR, USA). Slides were mounted in ProLong Diamond Antifade Mountant (Life Technologies, Thermo Fisher Scientific, Carlsbad, CA, USA). For immunofluorescence analysis, endocardial biopsies were imaged using LSM 501 confocal microscopy equipped with a digital camera (Zeiss, Jena, Germany).

### 2.7. Statistical Analysis

For continuous variables, descriptive statistics were provided (number of available observations, mean, and standard deviation), while the median (interquartile range) was used for non-normal data. Categorical data were described as numbers (percentage). The normal distribution of all continuous variables was checked using visual methods (Q-Q plot and histogram) and through the significance test (Kolmogorov–Smirnov normality test and Shapiro–Wilk’s test). Comparisons among groups were performed using Pearson’s bivariate test and chi-square tests for categorical covariates, one-way analysis of variance for continuous normally distributed covariates, and a non-parametric Kruskal–Wallis test for non-normally distributed continuous variables. For all tests, a *p*-value < 0.05 was considered statistically significant. The relationship between the number of NGCs and age was assessed using Pearson’s correlation coefficient (r) and the *p* value. All statistical analyses were performed using R statistical analysis software (version 2023.09.0).

## 3. Results

Two hundred and thirteen patients with a conclusive diagnosis of acute myocarditis were enrolled in our study [mean age: 46.8 ± 15.9; female: 61 (28.6%)]. As reported in Table 1, the incidence of hypertension was significantly different between groups [Group 1: 31 (34.8%) vs. Group 2: 38 (54.3%) vs. Group 3: 31 (57.4%); *p*-value 0.01]; no significant difference between groups was observed in terms of clinical presentation or echocardiographic or CMR findings.

CMR documented in all patients abnormal myocardial areas of T2 signaling and LGE enhancement in a non-ischemic pattern indicative of myocardial edema and necrosis compatible with acute myocardial inflammation (Lake Louise criteria).

All patients met the histological criteria for active lymphocytic myocarditis (Dallas criteria implemented with immunohistochemical characterization of inflammatory cells) at endomyocardial biopsy.

No major complications were reported following invasive cardiac studies, including cardiac biopsy.

Viral genomes were detected through PCR in 34 patients. Specifically, an adenovirus was found in 12 cases, an enterovirus in 8, influenza virus A in 7, Epstein–Barr virus in 5, and human herpesvirus in 2.

**Figure 1:**
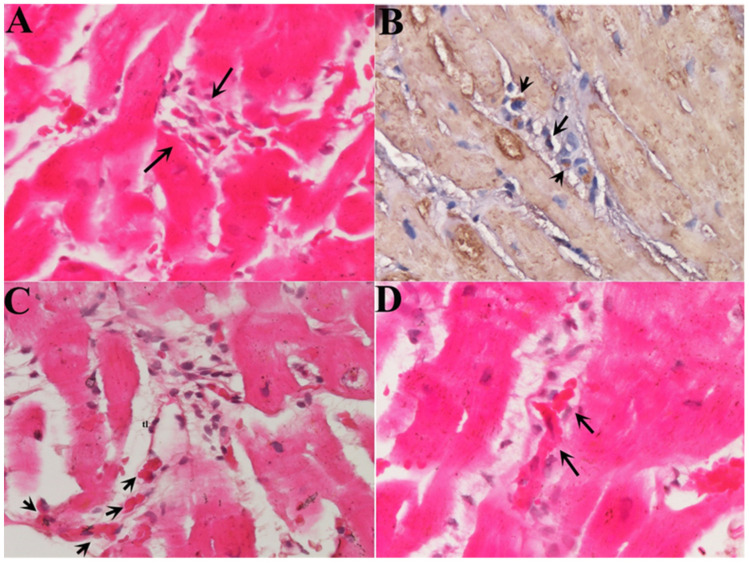
Homing and integration of NGCs into inflamed myocardium. (**A**) Several cells resembling small myocytes (arrows) approach an area of cardiomyocyte necrosis caused by lymphocytic myocarditis (H&E 400×). (**B**) Immunohistochemistry for alpha-sarcomeric-actin (400×) shows the presence of contractile material in the small cells. (**C**) Multiple NGCs are lining up (arrows) to enter an empty cardiomyocyte, crossing its cell membrane (H&E 400×). (**D**) NGCs (arrows) are growing inside of a cardiomyocyte that has undergone a myocytolytic process (H&E, 400×).

Small cells (Figure 1A) (with a diameter at the nuclear level of 6.6± 3.34 µm compared with adult myocytes of 22.5 ± 3.11 µm) were observed in the setting of necrotic areas identifiable as NGCs. These small cells express contractile material in the cytoplasm (arrows), confirmed to be small myocytes (Figure 1B)

**Figure 2:**
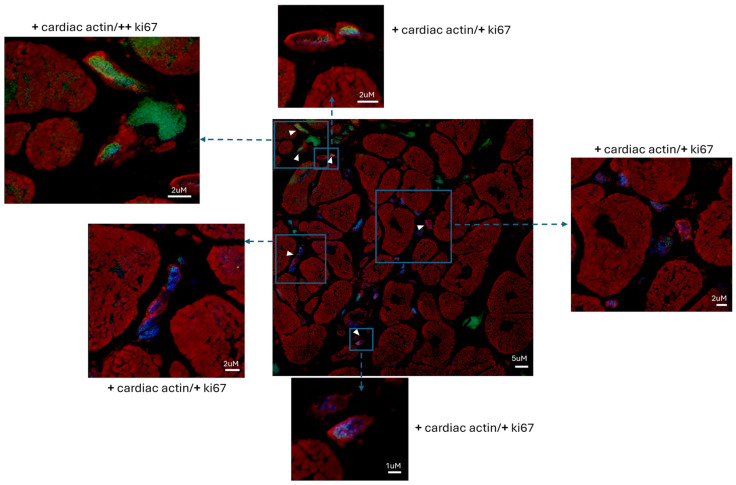
Myocarditis induces the formation of proliferating NGCs’ α-sarcomeric-expressing cells in the human left ventricle. Newly formed cells positive for α-sarcomeric actin (red fluorescence) expressed Ki-67 (green fluorescence), as indicated by the arrowheads. Red fluorescence indicates α-sarcomeric actin immunostaining; green fluorescence indicates Ki-67; blue fluorescence indicates DAPI staining of nuclei. Newly formed cells positive for α-sarcomeric actin (red fluorescence) expressed Ki-67 (green fluorescence), as indicated by the arrowheads. Bar = 5 µm (inserts: magnified version; bar = 2 µm or 1 µm).

NGCs expressed Ki-67 in the nuclei and alpha-sarcomeric actin in the cytoplasm (Figure 2). Ki-67 is a nuclear marker of cell proliferation that is no longer detectable in adult differentiated cells, while alpha-sarcomeric actin is a staining that recognizes contractile material characteristic of myocytes.

**Figure 3:**
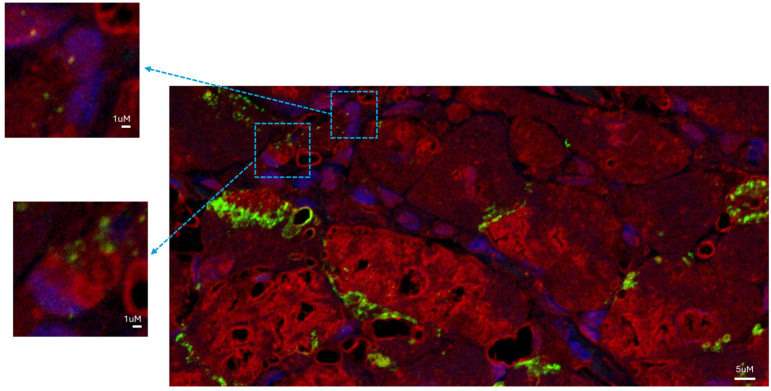
Newly generated cardiomyocytes in the human left ventricle were functionally competent. NGCs expressed connexin-43, detected as punctuate staining (green fluorescence) in the gap-junctional regions between cardiomyocytes in the left ventricular tissue. Newly formed α-sarcomeric-expressing cells expressed connexin-43. Red fluorescence indicates α-sarcomeric actin immunostaining; green fluorescence indicates connexin-43; blue fluorescence indicates DAPI staining of nuclei. Bar = 5 µm (inserts: magnified version; bar = 1 µm).

Connexin-43 is a molecule that is part of the structure of the gap junction responsible for electrical coupling between cardiomyocytes. Connexin-43 was abundantly distributed at the junctions between mature cardiomyocytes, but it was also detected as a punctuate staining at the surface of some newly formed cardiomyocytes (Figure 3).

**Figure 4:**
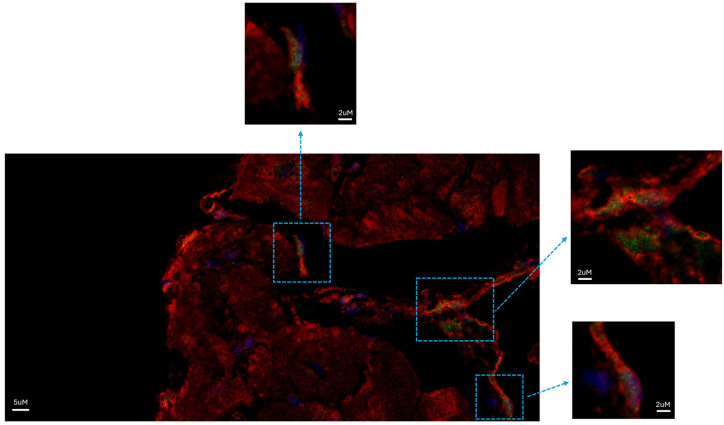
STAT3 was activated in NGCs of patients diagnosed with myocarditis. NGCs expressed pSTAT3 in the nuclei (green fluorescence). Red fluorescence indicates α-sarcomeric actin immunostaining; green fluorescence indicates pSTAT3; blue fluorescence indicates DAPI staining of nuclei. Bar = 5 μm (inserts: magnified version; bar = 2 µm).

Immunofluorescence also revealed the translocation of STAT3 to the nucleus in several NGCs, as evidenced by nuclei’s expression of pSTAT3 (Y705) (Figure 4). The translocation of pSTAT3 has been correlated with cell proliferation.

**Figure 5:**
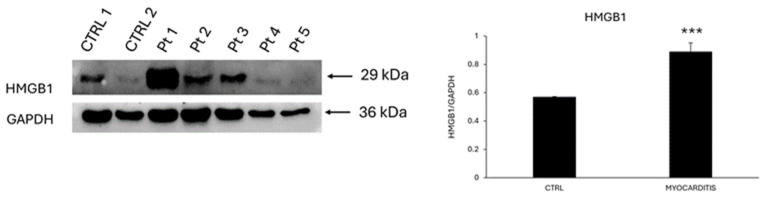
HMGB1 expression is up-regulated in myocarditis. Western blot analysis showing the expression of HMGB1 in LV endomyocardial biopsies of myocarditis compared to controls (CTRL). The same filter was probed with anti-GAPDH pAb to show equal loading. Left panel: a representative Western blotting of three replicates is shown. Marker: lane 1 = CTRL 1, lane 2 = CTRL 2, lane 3 = pt 1, lane 4 = pt 2, lane 5 = pt 3, lane 6 = pt 4, and lane 7 = pt 5. Right panel: densitometric analysis of Western blot (mean values) from CTRL patients (n = 2) and patients with myocarditis (n = 5). Data are shown as means ± SEM. ***, *p* < 0.001.

Interestingly, the protein expression level of HMGB1, an alarmin that activates the IL-6/STAT3 signaling pathway, was significantly higher (1.6 folds; *** *p* < 0.001) in endomyocardial biopsies from patients diagnosed with myocarditis compared to controls (Figure 5).

**Figure 6:**
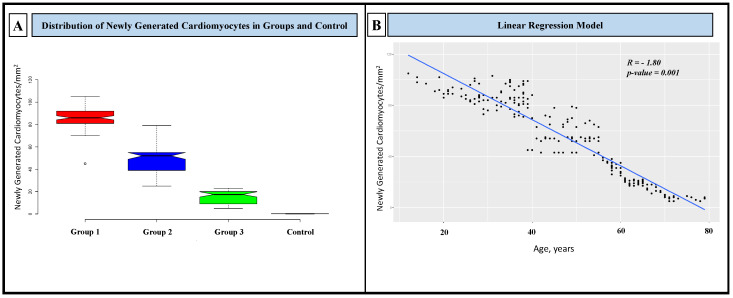
Distribution of newly generated cardiomyocytes in groups and controls and their correlation with age. (**A**) NGCs were statistically different in the three groups and between groups and controls (*p*-value between groups: 0.001). In the box plot are represented the median (line in the middle) and the middle “box” (1st–3rd quartile) of all values. The upper and lower whiskers represent scores outside of the middle 50%. (**B**) NGCs linearly decreased with age.

As far as the NGC numbers are concerned, in G1 (patients between 18 and 40 years), they were 86.3 ± 10.3/mm^2^; in G2 (those between 41 and 60 years), they were 50.4 ±13.8/mm^2^; and in G3 (those between 61 and 80 years), they were 15.1 ± 5.7/mm^2^. Control NGCs were 0.2± 0.2/mm^2^ (Figure 6A). The NGC number was not statistically different in viral or non-viral myocarditis. The value of NGCs was significant and inversely associated with age (R—1.80; *p*-value: 0.001) (Figure 6B); no other significant associations were reported.

In morphological terms, NGCs were in close contact with the scaffolds of empty cardiomyocytes derived from myocytolytic activity of perforins bound to toxic, CD3+, T lymphocytes. They were found, in sequential histological sections, to cross the cytoplasmic membrane and grow inside of the necrotic cell (Figure 1C,D).

## 4. Discussion

Improvement of cardiac dimensions and function in patients with acute myocarditis is common either in response to supportive measures (such as diuretics, ACE inhibitors, carvedilol, digitalis, gliflozins, etc.) and/or specific treatments, such as anti-viral and immunosuppressive agents. In particular, the BICC trial has demonstrated the ability of Beta-interferon to improve myocarditis promoted by entero- and adenovirus [15], while the TIMIC study reported the beneficial influence of immunosuppression on virus-negative inflammatory cardiomyopathy [16,17].

Nevertheless, complete cardiac recovery may occur spontaneously in up to 50% of cases with this affliction [1], suggesting an important contribution from natural pathways of repair. In this last regard, the mechanisms involved are not yet completely clarified, although they include the decline of operating noxa with a decrease in cell death and cardiomyocyte repair through resynthesis of the contractile protein and then myocyte proliferation. In a previous report [2], myocyte replication was reported to increase by 43% for Ki-67 and by 38% for MCM5-positive cells after 6 months of successful immunosuppression. However, the morphologic characterization of NGCs, their modality of integration in the inflamed myocardium, and their proliferative reserve with age have not been addressed so far.

Studying the histologic substrate of an extensive number of patients with acute myocarditis, we were able to systematically detect NGCs in the proximity of necrotic cells (Figure 1). They had small dimensions (6.6 ± 3.34 µm at the nuclear level) and they were recognizable for the cytoplasm containing α-sarcomeric actin positive material, a cell membrane expressing connexin-43, and positive nuclear immunostaining for Ki-67 and phospho-STAT3, denoting a proliferative state of cells.

Likely, cardiomyocyte-oriented stem cells, resident in the myocardium or released by bone marrow, might migrate to the site of cell necrosis following specific chemotactic stimuli, like HMGB1 [18]. Accordingly, we detected an up-regulation in the expression of this protein in the endomyocardial biopsies from patients with myocarditis compared to controls [19]. Our finding is supported by a preclinical study that adopted a mouse model of EAM. Specifically, Su and colleagues demonstrated that HMGB1 increased in the cardiac tissue and in the blood of EAM mice, and its blockage with an anti-HMGB1 antibody reduced infiltration of T helper-17 cells, serum levels of inflammatory molecules, and cardiac fibrosis. Cardiac fibroblast/myofibroblasts represented the source of the secreted HMGB1 that contributed to collagen deposition. Interestingly, the authors showed that HMGB1 could also promote the proliferation and migration of these cells. It is tempting to speculate that this proliferative effect exerted by HMGB1 toward cardiac fibroblasts/myofibroblasts might also include other cell types, such as resident stem cells.

In our study, we were able to demonstrate that NGCs colonize the damaged myocardium, crossing the scaffolds of necrotic myocytes (Figure 1) resulting from the perforating activity of toxic T lymphocytes. NGCs were able to grow inside of empty cells (Figure 1), realizing a perfect integration that preserves the structure and function of the inflamed myocardium. To obtain homing and integration of NGCs into the inflamed myocardium, it is likely that at least two pre-requisites are need: (1) preserved coronary circulation and (2) partial integrity of the plasma membrane of necrotic myocytes. Both of these requisites are lacking in human myocardial infarction, and this likely explains the lack of structural recovery in this clinical instance.

Importantly, our results suggest that myocarditis might promote regeneration at least partially by stimulating HMGB1/IL-6/STAT3-dependent NGC proliferation. This is in accordance with previous studies showing the ability of IL6/STAT signaling to induce cartilage regeneration mediated by cartilage stem/progenitor cells [7] and the key role of this signaling in reactive cardiomyocyte proliferation induced by myocardial injury in neonatal mice [4]. Moreover, it is well-known that HMGB1 propagates the inflammatory response through the induction and secretion of inflammatory cytokines and chemokines, including IL6, and promotes the activation and proliferation of stem/progenitor cells [20].

Furthermore, we analyzed the proliferative reserve of NGCs with age. We found a progressive decline of cardiomyocyte replication and then the ability to repair cardiac damage and dysfunction caused by myocarditis. Specifically, the group of patients aged 61–80 years had an NGC number of 15.1 ± 5.7/mm^2^ compared with 86.3 ± 10.3/mm^2^ among patients aged 18–40 years with the same degree of cardiac compromise (LVEF ≤ 30%). This suggests an increased risk of disability and death in aged patients undergoing acute myocarditis. Ultimately, the proliferative ability of NGCs in the context of acute cardiac inflammation seems independent of the cause, as we did not find a difference in NGC numbers between viral and non-viral myocarditis (*p* > 0.05).

### Limitations of the Study

The present study analyzes cardiomyocyte regeneration in acute human myocarditis. What occurs in patients with chronic myocarditis or other forms of myocardial injury and cell death, like cell apoptosis, has yet to be established. In particular, the extent of the cardiomyocyte proliferative reserve, and the importance of maintaining cardiomyocyte scaffolds for the integration of NGCs, have to be clarified. Finally, the fascinating molecular mechanisms that are at the basis of the homing and integration of NGCs, allowing for replacement of the necrotic myocytes, are still to be defined.

In conclusion, NGCs are constantly recognizable in acute human myocarditis. Low cell dimensions and molecular signals of active myocyte replication as Ki-67 and STAT3 are the main indicators. Their number declines with age. Their integration within necrotic myocytes allows for the preservation of the cardiac structure and function.

## Figures and Tables

**Table 1:** Baseline characteristics and risk factors of the study population.

Demographics	Group 1 (n = 89)	Group 2 (n = 70)	Group 3 (n = 54)	*p*-Value
Age, yrs	31.2 ± 7.1	50.9 ± 5.6	67.2 ± 4.8	**<0.001**
Female	31 (34.8)	15 (21.4)	15 (27.8)	0.18
Arterial Hypertension	31 (34.8)	38 (54.3)	31 (57.4)	**0.01**
Diabetes	3 (3.4)	8 (11.4)	5 (9.6)	0.14
Atrial Fibrillation	11 (12.4)	12 (17.1)	14 (25.9)	0.11
***Clinical manifestation***				
Heart Failure	80 (89.9)	65 (92.9)	51 (94.4)	0.59
Electrical Instability	9 (10.1)	5 (7.1)	3 (5.6)	0.59
***Echocardiographic parameters***				
LVEF, %	42.1 ± 15.9	38.2 ± 14.1	38.0 ± 12.4	0.16
LVEDV, mL/m^2^	86.7 ± 31.0	99.5 ± 39.9	92.2 ± 26.9	0.12
LVESV, mL/m^2^	51.3 ± 27.3	60.9 ± 35.1	56.9 ± 23.2	0.17
TAPSE, m	20.7 ± 3.8	20.7 ± 4.5	20.1 ± 4.0	0.69
***MRI parameters***				
LVEF, %	44.7 ± 16.1	39.0 ± 16.0	39.3 ± 15.9	0.10
LVEDV, mL/m^2^	97.1 ± 34.6	111.3 ± 42.8	98.6 ± 34.7	0.09
LVESV, mL/m^2^	57.9 ± 36.0	67.9 ± 40.8	61.5 ± 35.5	0.48
LGE	66 (74.2)	57 (81.2)	39 (72.2)	0.90
Myocardial Edema	51 (57.3)	45 (64.3)	36 (66.7)	0.43

Values are n (%) or mean ± standard deviation. LGE: late gadolinium enhancement; LVEF: left ventricular ejection fraction; LVEDV: left ventricular end diastolic volume; LVESV: left ventricular end systolic volume; TAPSE: tricuspid annular plane excursion.

## Data Availability

The datasets used and analyzed during the current study are available from the corresponding author upon reasonable request.

## Abbreviations

CMR	Cardiac magnetic resonance
EAM	Experimental autoimmune myocarditis
EDTA	Ethylenediaminetetraacetic acid
H&E	Hematoxylin and eosin
HMGB1	High mobility group box 1
IL6	Interleukin 6
Jak1	Janus kinase signal transducer 1
LGE	Late gadolinium enhancement
LVEF	Left ventricular ejection fraction
MCM5	Mini-chromosome maintenance 5
α-MHC	Alpha-myosin heavy chain
NGCs	Newly generated cardiomyocytes
PBS	Phosphate-buffered saline
Rln3a	Relaxin 3A
STAT3	Signal transducer and activator transcription 3
